# Perceived usefulness of a distributed community-based syndromic surveillance system: a pilot qualitative evaluation study

**DOI:** 10.1186/1756-0500-4-187

**Published:** 2011-06-14

**Authors:** Blaine Reeder, Debra Revere, Donald R Olson, William B Lober

**Affiliations:** 1 Biobehavioral Nursing and Health Systems School of Nursing, Box 359442 University of Washington, Seattle, WA 98195, USA; 2 Department of Health Services School of Public Health, Box 354943 University of Washington, Seattle, WA 98195, USA; 3 International Society for Disease Surveillance 26 Lincoln Street, 3 Brighton, MA 02135, USA; 4 Medical Education and Biomedical Informatics School of Medicine, Box 357240 University of Washington, Seattle, WA 98195, USA

## Abstract

**Background:**

We conducted a pilot utility evaluation and information needs assessment of the Distribute Project at the 2010 Washington State Public Health Association (WSPHA) Joint Conference. Distribute is a distributed community-based syndromic surveillance system and network for detection of influenza-like illness (ILI). Using qualitative methods, we assessed the perceived usefulness of the Distribute system and explored areas for improvement. Nine state and local public health professionals participated in a focus group (*n = 6*) and in semi-structured interviews (*n = 3*). Field notes were taken, summarized and analyzed.

**Findings:**

Several emergent themes that contribute to the perceived usefulness of system data and the Distribute system were identified: 1) *Standardization: *a common ILI syndrome definition; 2) *Regional Comparability: *views that support county-by-county comparisons of syndromic surveillance data; 3) *Completeness: *complete data for all expected data at a given time; *4) Coverage: *data coverage of all jurisdictions in WA state; 5) *Context: *metadata incorporated into the views to provide context for graphed data; 6) *Trusted Data*: verification that information is valid and timely; and 7) *Customization: *the ability to customize views as necessary. As a result of the focus group, a new county level health jurisdiction expressed interest in contributing data to the Distribute system.

**Conclusion:**

The resulting themes from this study can be used to guide future information design efforts for the Distribute system and other syndromic surveillance systems. In addition, this study demonstrates the benefits of conducting a low cost, qualitative evaluation at a professional conference.

## Introduction

Distribute is a community-based, population-level public health information system for syndromic influenza-like illness (ILI) surveillance that displays aggregated, de-identified, public health surveillance data collected from emergency departments (EDs) by state and local health jurisdictions [1]. Distribute was first organized by the International Society for Disease Surveillance (ISDS) in 2006 as a proof of concept. With support from the Markle Foundation and the United States Centers for Disease Control and Prevention (CDC), Distribute grew from participation by 8 state and large metropolitan health jurisdictions, representing summarized data on 10% of all US emergency department (ED) visits, to a nation-wide system that currently receives data from 43 health jurisdictions that represents over 50% of the US population and summarizes more than 35% of all ED visits nationwide. Distribute currently has participation from all ten Health and Human Services (HHS) surveillance regions, includes data from over one million ED visits each week, and displays updated visualizations of ILI trends in the US on Public and Restricted access web sites.

Distribute serves as an example of a new paradigm in the collection and sharing of public health surveillance data [2]. The roots of automated syndromic surveillance systems began just prior to the 2001 Anthrax attacks [3] with systems which automatically classify clinic visits and other data according to loose "syndromic" criteria and present graphic and statistical views of summarized data based on counts of those visits, and their numbers in proportion to population and denominators derived from utilization of health care services. The literature describes both the early experience and growth of these systems [4] and their evolving design and implementation [5]. With the development of health information exchanges (HIEs), and of methods for the structuring of, and access to, regional data across multiple health care systems, public health gained access to larger sources of both visit level and summarized "syndromic" data [6]. In part, Distribute developed as a way for health departments to share and compare these summarized, syndromic data, regardless of whether those data were obtained from integration of data from individual providers or hospitals, or from a single large hospital network or HIE.

In the early development of the Distribute project, the apparent benefits of mandated standards for syndrome definitions were weighed against two often overlooked issues: barriers to entry and ability to compare data across jurisdictions. The need to adopt a mandated standard prior to joining the network created a potential technical barrier that could delay or prevent interested jurisdictions from participating. In addition, although mandated standard syndrome definitions could improve data comparison on average across the whole network, there was concern on the part of project participants that this practice might decrease accuracy and utility locally. That is, a local definition of a syndrome might best reflect local variations in coding or clinical practice that were reflected in the data, and might most accurately reflect the underlying disease being tracked. To address these issues, the Distribute project adopted the use of two separate syndromes: 1) a more narrow and specific definition, following a traditional clinical definition of ILI, and 2) another more sensitive definition, as a broad febrile, respiratory and influenza-like syndrome [7]. In a preliminary comparison, two Distribute participating sites shared local coding of their narrow and broad ILI syndrome definitions and applied each other's definitions to their own local data. The pilot findings suggested that data using locally applied syndromes were better correlated with population-level viral surveillance data [8,9].

### Utility and Usability

Utility and usability issues are related and often difficult to separate in the evaluation of information systems [10,11]. Utility, or perceived usefulness, refers to the extent to which an information system or its output provides benefit or value [10-12]. Usability, or perceived ease of use, refers to the degree of effort required to use an information system or its output [10-12]. Many international standards for system design conflate usability and utility, incorporating aspects of utility and usability in a single definition [13]. Because this project is not an interaction study, we focus on the utility, or perceived usefulness, of the Distribute system and its data outputs while acknowledging that usability contributes to utility.

Current initiatives of the Distribute project place a high priority on improving the utility and usability of the information system and extending functionality to support public health decision-making and practice. Qualitative methods are important in the evaluation of health information systems [14-16]. It is important to engage practitioners in a discussion of their needs and proposed system features to mitigate common informatics risk factors for failed system adoption [17-19]. Following the idea that "the simplest way to assess usefulness is to ask those involved in public health practice"[20], we engaged epidemiologists and other public health practitioners in a pilot study to collect quality improvement feedback for the Distribute system. This pilot study was undertaken to inform the design of a larger quality improvement investigation by including participants who were current members of the Distribute community of practice and those interested in learning more about the Distribute system.

### System Description

The objective of Distribute is to collect, analyze, and display ILI surveillance data from across the United States. Another objective is to provide ways to compare the progression of outbreaks of infectious disease between regions, and to enhance communication between health jurisdictions. Distribute displays summary level data from state and local health department ED surveillance systems in two views: Public and Restricted. The Public site provides public access graphs of weekly trends in ratios of ILI syndromes to all ED visits. The restricted site requires secure authentication for access and provides greater granularity in the time series data, primary counts as well as ratios, multiple syndromes, information about data upload history and transmission details, data timeliness, detailed visualizations based on user-specified queries and metadata that includes background information about each data provider and details of syndrome definitions. This secure view of information about all data-providing jurisdictions is available to each participating data provider on the restricted site. Distribute data providers upload data from existing surveillance systems, such as ESSENCE [21], EARS [22], RODS [23]and BioSense [24], at local and state health jurisdictions along with provider-specified syndrome definitions. Table 1 shows selected metadata elements available to data providers with restricted site access.

**Table 1:** Selected metadata elements from the Restricted site of the Distribute system

Element	Description	Possible Values
*Geo Type*	The organizational area view of the data	City, State, Region, Federal Region
*Preferred Syndrome*	The syndrome definition preferred by the data providers for display of their uploaded data	Defined by data provider. Examples: *ILI-broad, ILI-narrow, GI-broad, GI-narrow, Temperature, Disposition*
*Available Indicators*	All syndrome definitions for data submitted by data providers	Defined by data provider. Examples: *ILI-broad, ILI-narrow, GI-broad, GI-narrow, Temperature, Disposition*
*Syndrome Descriptions*	Details of syndrome definitions for data submitted by providers	Defined by data provider
*Stratification Descriptions*	The criteria by which data are stratified	Defined by data provider. Examples: *age group, zip3, temperature, disposition*
*Facilities Sending Data*	Enumerated list of health care facilities that submit data to be aggregated to the data provider	Variable by number of participating facilities in the jurisdiction of the data provider
*Facilities in Jurisdiction*	Enumerated list of health care facilities in the jurisdiction of the data provider	Variable by number of total facilities in the jurisdiction of the provider
*Visit Types*	Types of facilities for which visit data are submitted	Variable by data provider. Typically *Emergency Departments *and *Urgent Care *facilities
*Typical Record Count*	Description of expected record volume based on historical patterns	Variable by data provider
*Population Coverage*	Description of the population and the number and type of health care facilities in the jurisdiction	Variable by data provider
*Local System Description*	Description of the local surveillance system	Examples: *ESSENCE II, EARS, RODS, BioSense*
*Data Source History*	Description of the onset date of data availability	Variable by data provider
*Last Data Point Visualized*	Description of the last available date for which data are available	Variable by data upload pattern
*Days Old*	Calculated value based on the last available date for which data are available	Variable by data upload pattern
*Last Upload Date*	Description of the last date of data upload	Variable by data upload pattern
*Days Ago*	Calculated value based on the last date of data upload	Variable by data upload pattern
*Upload Frequency*	Description of the frequency with which a data provider typically uploads data	Variable by data provider

## Methods

### Setting

This study was conducted at the Washington State Public Health Association (WSPHA) Joint Conference on Health on October 11-12, 2010 in Yakima, WA USA. The conference is an annual meeting of public health practitioners that includes participants from the Washington State Department of Health and local health jurisdictions in the State of Washington. The study protocol received approval from the University of Washington Institutional Review Board.

### Participants

A total of nine public health practitioners participated, representing state- (*n = 5*) and county- (*n = 4*) level public health organizations. Six participants attended a focus group and three participants engaged in brief interviews. Seven participants were current epidemiologists, health officers or other public health practitioners while two participants were former epidemiologists or health officers. Regarding prior use and/or familiarity with Distribute, four participants had never seen or used Distribute and of the five who were familiar with Distribute, only two participants had access to the Restricted site. All participants were familiar with how surveillance data are used for public health purposes. Table 2 shows a breakdown of study participants by role and data collection method.

**Table 2:** Study participants by role, agency level, familiarity with Distribute and method of data collection

Participant	Role	Agency Level	Familiar with Distribute?	Method of Data Collection
WA01	Health Officer	County	No	Focus Group
WA02	Epidemiologist	County	No	Focus Group
WA03	Epidemiologist (Former)	County	Yes	Focus Group
WA04	Communicable Disease Director	County	Yes	Interview
WA05	Epidemiologist	State	Yes	Interview
WA06	Public Health Planner	State	No	Focus Group
WA07	Epidemiologist	State	Yes	Focus Group
WA08	Epidemiologist	State	Yes	Focus Group
WA09	Health Officer (Former)	State	No	Interview

### Data Collection

We employed qualitative methods [25-27] to capture participants' perceived usefulness of data, data visualizations and features of the Distribute system and solicit quality improvement feedback from participants: 1) a focus group discussion of the Distribute system during a scheduled presentation and 2) three brief, semi-structured interviews with conference attendees who fit the profile of the target user group. Following a brief project background, participants were shown different views of graphed data for US Department of Human and Health Services (HHS) Regions from the Public site and Restricted site comparisons of graphed data from data providers in the Pacific Northwest for periods of time covering the 2009-2010 H1N1 and influenza seasonal time periods back to March 2009. Participants were asked to respond about perceived usefulness of views of graphed data and to discuss how they use surveillance data in their work. Focus group and interview participants were asked the same types of questions. Those familiar with Distribute were asked questions about their perceptions of the system. Table 3 shows the types of questions asked of participants.

**Table 3:** Types of questions asked of focus group and interview participants

• What does this graph tell you?
• What might be missing from this graph?
• Would graphed data like these have been useful during the 2009-2010 influenza A/H1N1 season?
• Would graphed data like these be useful during seasonal influenza time periods?
• Is this a good way to display the data?
• How might this graph be more useful?
• What do you like about Distribute?
• How useful is Distribute?
• How could Distribute be better?

**Figure 1:**
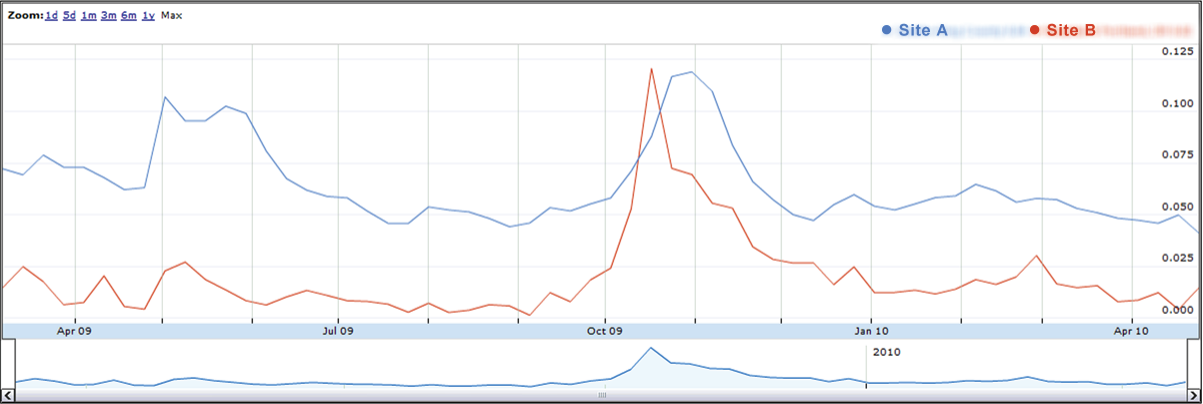
**De-identified jurisdiction comparison showing the emergence and return of pandemic A/H1N1 influenza, March 2009-April 2010**.

Figure 1 shows a comparison graph used during data collection. The data-providing jurisdictions in the graph are de-identified for publication. The graph shows ILI visit time-series ratios of syndromic ED surveillance data during March 2009 through April 2010 from two Northwest US jurisdictions (Sites A and B) participating in Distribute. The ratios presented are ILI syndrome visits over total visits using syndrome definitions that are jurisdiction-specific and not standardized. While the absolute ILI levels are not directly comparable, the timing and relative magnitude show ILI trends representing the emergence and early spring wave of the influenza A/H1N1 pandemic and its autumn 2009 return.

### Data Analysis

Notes taken by Distribute team members during the focus group and interviews were summarized and analyzed to identify patterns and themes [28,29]. Study notes were stripped of identifying information before analysis. Conference attendees were referred to by role and an assigned study code. Specific data extracted from study notes pertained to opinions of conference attendees about usefulness, suggested features and other recommendations for improvement of the Distribute system. Themes were identified from focus group and interview notes by grouping similar responses and creating names and descriptions of the groupings [26,28].

## Results

Overall, participants were engaged and positive about Distribute as a community-based network and syndromic surveillance system. Data analysis resulted in the emergent themes, which are displayed in Table 4 along with brief descriptions of each theme.

**Table 4:** Themes that contribute to information system and data usefulness

Theme	Description
Standardization	A common fixed case definition of influenza-like illness (ILI)
Regional Comparability	Division of views into different regions to aid in comparisons
Completeness	Extent to which the expected data are provided
Coverage	Extent to which data are representative of populations to facilitate generalizations
Context	Group or macro-level variables that frame the data; also referred to as metadata
Trusted Data	Knowledge of the extent to which the data presented are valid and timely
Customization	Availability of data and processing features to meet the surveillance needs of the local or state health jurisdiction

With regard to standardization, participants recognized a need for a common influenza-like illness (ILI) syndrome definition in order to make comparisons between data sets more meaningful and relevant. For regional comparability, participants wanted to see views of different regions to aid in comparisons. In particular, they expressed a desire for views that support county-by-county comparisons of syndromic surveillance data, separate regional views of Western Washington and Idaho and views by preparedness regions as an alternative if representative views of HHS Regions were unavailable.

The completeness theme is described by participant desire for completeness in the data sets submitted from each data provider. Coverage refers to participant desire to know that data are representative of a given population in order to generalize findings across the population. In particular, participants expressed a need for data coverage of all Washington State. Participants noted that during the second wave of H1N1, the eastern side of the state, which includes one-third of the population, initially saw two-thirds of all cases (consistent with the graph of data for Eastern Washington in Distribute). One participant noted minimal use of Distribute due to lack of close neighbors for comparison. Context refers to the expressed need for metadata incorporated into views to facilitate understanding of graphed data. Participants suggested inclusion of the number of hospitals, emergency departments, patients, data providers and denominators for the total number of ED visits as contextual information in the graphed data views.

Participants expressed a need for trusted data, that is, confidence that data are valid and timely. They wanted to know that data were verified through defined quality assurance processes that are conducted on a regular basis. Participants reported that these data are useful for consistent events such as seasonal influenza and local data could be used to declare a local epidemic. Consistent, reliable data were cited as more useful to stand-down from an emergency than to issue an initial alert; participants hypothesized that during H1N1 the data in Distribute might have been more useful after confirmation of an actual event occurrence. Customization of available data and data processing capabilities to meet the surveillance needs of each local and state health jurisdiction was requested by participants. The ability to overlay graphs with other graphs and create labels on request was envisioned as a useful feature.

Participants acknowledged the value of the Public site as a tool to view national trends. One participant cited the need for a surveillance system with a low-impact training cost that anyone can use and that is largely automated to minimize maintenance. Requests for additional data viewed as overlays to graphs included:  metadata already available elsewhere in the system, county school absenteeism rates and a state view that includes data from all clinics in the Group Health Cooperative health care system. An additional result of the focus group was the expressed desire to participate as a data contributor by one participant from a county-level health jurisdiction.

## Limitations

The limitations of this pilot study include its restricted time frame for data collection and the regional population from which the sample is drawn.

## Conclusion

Our results suggest themes that can be used to guide future evaluation and design iterations to improve support for public health surveillance. These results are important for improvements to syndromic surveillance of influenza-like-illness in the Distribute system but can also help improve syndromic surveillance efforts overall, regardless of the disease or surveillance system. For example, gastrointestinal (GI) indicators are currently being piloted in the Distribute system as a demonstration of system extensibility for surveillance of other diseases. Themes from this qualitative evaluation study can inform GI syndromic surveillance efforts as they are expanded within Distribute or any other surveillance system. These themes should be further explored by including public health practitioners in information design efforts. In addition, this study demonstrates that the application of qualitative methods in an "evaluation of opportunity" at a public health practice gathering can be a simple way to solicit feedback for the improvement of a working public health information system. Lastly, we found that efforts of this type can be useful in recruiting new users to participate in the system and expand the community of practice.

The community-based approach employed in the Distribute project focuses on data use and has resulted in convergence toward a recognized need for a common influenza-like illness (ILI) syndrome definition to compare data sets across jurisdictions among the Distribute community of practice. The findings of this pilot study are consistent with this trend. However, to maintain local utility of data, existing data providers need not, and should not, abandon prior syndrome definitions, but rather should submit an additional common definition while continuing to send data aggregated by existing syndrome definitions that have local meaning.

Syndromic surveillance data, if available, are used by public health practitioners as early indicators of influenza outbreaks within their own jurisdictions and adjacent health jurisdictions. These data are used in conjunction with other data sources, such as laboratory results, to triangulate disease prevalence. To aid decision-making for interventions that help contain outbreaks, improved data access and visualizations for syndromic surveillance data are needed. The context of how data are used for individual tasks is important [30,31] and data quality cannot be assessed independent of the people who use them [32]. Information systems are part of the contexts of use for data; the utility and usability of these systems are factors in the utility and usability of data [13,33,34]. Three contexts of use for syndromic surveillance information systems - routine, anticipated threat and present threat - have been recognized as key inputs to tasks for analysis and characterization of syndromic surveillance data for decision-making [35]. In addition, our pilot results indicate contextual information - metadata related to hospitals, patients, data providers, ED visit counts, etc. - provides meaning of syndromic surveillance data to epidemiologists.

To better understand contexts of information system and data use, future efforts should identify the specific ways in which epidemiologists and others use metadata to discern meaning from data, the best ways to include annotations in data visualizations and different ways to display information for population health surveillance. Interviews with a larger number of participants will help refine the specific meanings of our themes, gauge reactions to anticipated results from common ILI syndrome definition efforts and explore specific needs around regionalization and other identified themes. Future work to engage a more geographically diverse population of participants will help validate these results outside the pilot region of Washington State. This future work will be informed by pilot results in two related areas: 1) usability studies to improve the design of Distribute as an information resource that an epidemiologist might check before making a phone call to a colleague in a different jurisdiction or region and 2) utility studies to assess the value of Distribute to participants, their organizations and community population health outcomes.

## Competing interests

All authors declare financial support from the International Society of Disease Surveillance. The authors declare that they have no other competing interests.

## Authors' contributions

BR conceived of and implemented the study design with DR, acted as investigator/observer, collected and analyzed data and authored the overall manuscript. DR conceived of and implemented the study design with BR, authored the protocol, acted as investigator, collected and analyzed data and authored the overall manuscript. DRO contributed expertise and manuscript content related to the Distribute project and common ILI definitions. WBL contributed expertise and manuscript content about informatics and syndromic surveillance. All authors read and approved the final manuscript.
